# Establishment and evaluation of nomogram for predicting intraventricular hemorrhage in neonatal acute respiratory distress syndrome

**DOI:** 10.1186/s12887-023-03853-1

**Published:** 2023-01-28

**Authors:** Nurbiya Arkin, Yanmei Wang, Le Wang

**Affiliations:** Department of Neonatology, The First Affiliated Hospital of Xinjiang Medical University, 830054 Urumqi, Xinjiang China

**Keywords:** Intraventricular hemorrhage (IVH), Neonatal acute respiratory distress syndrome (ARDS), Risk factors, Nomogram, Prediction model

## Abstract

**Background:**

Intraventricular hemorrhage (IVH) is the most common type of brain injury in newborns, especially in newborns with Neonatal acute respiratory distress syndrome (ARDS). IVH can cause brain parenchyma damage and long-term neurological sequelae in children. Early identification and prevention of sequelae are essential. This study aims to establish a predictive nomogram for the early prediction of IVH in newborns with ARDS.

**Methods:**

From 2019 to 2021, we collected data from 222 infants diagnosed with ARDS in the Department of Neonatology, First Affiliated Hospital of Xinjiang Medical University. Infants have been randomly assigned to the training set (*n* = 161) or the validation set (*n* = 61) at a ratio of 7:3. Variables were screened using the Least Absolute Contract and Selection Operator (LASSO) regression to create a risk model for IVH in infants with ARDS. The variables chosen in the LASSO regression model were used to establish the prediction model using multivariate logistic regression analysis.

**Results:**

We recognized 4 variables as independent risk factors for IVH in newborns with ARDS via LASSO analysis, consisting of premature rupture of membranes (PROM), pulmonary surfactant (PS) dosage, PH^1^ and Arterial partial pressure of oxygen (PaO_2_^1^). The C-Index for this dataset is 0.868 (95% CI: 0.837–0.940) and the C index in bootstrap verification is 0.852 respectively. The analysis of the decision curve shows that the model can significantly improve clinical efficiency in predicting IVH. We also provide a website based on the model and open it to users for free, so that the model can be better applied to clinical practice.

**Conclusion:**

In conclusion, the nomogram based on 4 factors shows good identification, calibration and clinical practicability. Our nomographs can help clinicians make clinical decisions, screen high-risk ARDS newborns, and facilitate early identification and management of IVH patients.

## Introduction

IVH is the most common type of brain injury in neonates, especially in neonates with acute respiratory distress syndrome (ARDS) [1]. In the past decades, the increase in the rate of preterm birth has kept the total incidence rate of intraventricular hemorrhage high [2, 3]. IVH is still a major problem in the modern global neonatal intensive care unit (NICU). The importance of IVH is not only related to its high incidence rate but also related to its associated complications. The complications of IVH include periventricular leukomalacia [4], neurodevelopmental retardation [5, 6] and cerebral palsy [7]. Even low-level IVH is associated with long-term neurodevelopmental consequences, including intellectual disability and cerebral palsy [8–10]. Many factors have been identified as risk factors for IVH, including low gestational age, respiratory distress syndrome, prenatal non-exposure to steroids, intrauterine infection, hypoxia, hypercapnia, premature rupture of membranes, acidosis and sepsis, etc. [11–13]. Premature delivery and respiratory distress syndrome are the two most important risk factors for IVH. A large amount of evidence shows that [14–16], a considerable number of IVH cases occur in the first few hours of life. Brain ultrasound is the first choice for diagnosis, but due to the subjective influence and the limitations of lower-level hospital facilities and personnel, children may be difficult to perform early examinations. Therefore, it is important to determine the factors that have played a role in prenatal and early neonatal life, which can reduce the risk of these complications. However, so far, there are few studies on the influencing factors of IVH in children with ARDS, and there is no perinatal risk prediction model to predict the occurrence of IVH in patients. The nomogram transforms the complex regression equation into a visual graph, which makes the results of the prediction model more readable and has a higher use value, and provides clinicians with information related to management and treatment [17]. Therefore, in this retrospective research, we plan to develop and validate a simple and reliable nomogram to evaluate the risk of IVH in children with ARDS and provide recommendations for disease assessment, treatment, and complication prevention.

## Methods

This study aimed to retrospectively analyze the data of ARDS neonates hospitalized in the First Affiliated Hospital of Xinjiang Medical University. The research scheme was approved by the Ethics Committee of the First Affiliated Hospital of Xinjiang Medical University, and the individual agreement for the retrospective analysis was abandoned.

### Patients and data collection

The subjects were 222 newborns diagnosed with ARDS in the neonatal intensive care unit from October 1, 2019, to October 1, 2021. Depending on whether or not IVH occurred, patients were split into case groups and control groups. Data on neonatal characteristics (gender, gestational age, birth weight, length, head circumference, Apgar 1 min score), maternal characteristics (Uterine contraction, PROM, multiple births), blood gas analysis indicators (first and second Arterial blood gas after birth: PH, PaO_2_, PaCO_2_, BE), and neonatal intervention (use of PS, PS dosage(≤ 100 mg/kg or >100mg/kg), Invasive ventilation) were collected from medical records.

### Inclusion and exclusion criteria

The diagnosis of ARDS conforms to the definition of Montreux. All newborns diagnosed with ARDS are eligible if they meet the inclusion and exclusion criteria. Inclusion criteria: (1) dyspnea occurred within 24 h after birth and assisted ventilation was required; (2) The age of admission is less than 1 day; (3) Complete blood gas and ultrasonic examination within 24 h; Exclusion criteria: (1) patients with congenital heart disease, chromosome abnormality, brain structural malformation, and other congenital abnormalities and intrauterine cerebral hemorrhage; (2) incomplete data; (3) The respiratory function affected by other serious diseases of the system (such as respiratory distress syndrome (RDS) and neonatal transient tachycardia (TTN)) was excluded. Montreux standard [18]: The diagnosis of neonatal ARDS should meet the following requirements at the same time : ① the onset of the disease, and the acute attack (within 1 week) after identifying or suspicious inducements (asphyxia, choking, meconium aspiration, infection, etc.); ② NRDS, transient tachypnea of newborn (TTN) or dyspnea caused by congenital malformation were excluded; ③ Pulmonary imaging showed bilateral diffuse and irregular decreased light transmittance, exudation or white lung, which could not be explained by other reasons, such as local effusion, atelectasis, NRDS, TTN or congenital malformation; ④ The cause of pulmonary edema, which can not be explained by congenital heart disease, can be confirmed by cardiac ultrasound; ⑤ According to oxygen index, OI = FiO2 × Mean airway pressure (Paw) × 100/PaO2] Assessment of oxygenation disorder and disease severity: mild ARDS 4 ~ < 8, moderate ARDS 8 ~ < 16, severe ARDS ≥ 16.

### Definitions

The GE VIVID 7 color digital Doppler ultrasound diagnostic instrument was used to obtain standard coronal and sagittal images through the front fontanel, and the 4–8 MHz variable frequency phased array probe was used. The first cranial ultrasound examination was conducted within 24 h after birth. The severity of IVH was graded according to the improved Papile [19] grading system. Due to the limitation of ultrasound time, all included cases were mild IVH (Grade I, Grade II). The Montreux definition was used to make the diagnosis of neonatal ARDS [18]. PS dosage(≤ 100 mg/kg or >100mg/kg): According to the consensus of experts on the clinical application of pulmonary surfactant in newborns in China (2021) [20] and clinical experience, we chose this dose to divide different groups.

### Feature selection

The Least Absolute Shrinkage and Selection Operator (LASSO) is a regression analysis method that performs feature selection and regularization at the same time. It adds penalty parameters to the least squares regression to compress the estimated variables, thereby improving the prediction accuracy and interpretation of the model [21]. As a result, we used LASSO to determine the best predictive variables.

### Development and assessment of the nomogram

The variables identified by LASSO regression were incorporated into multiple logistic regression analyses to create a prediction model for the occurrence of IVH in newborns with ARDS. The model is established in the training set and then internally verified to create a nomogram with good calibration and discrimination capabilities. In the multivariate analysis, the variables with *P* ˂0.05 were included in the nomogram. The nomograph’s foundation is the scaling of each regression coefficient in multiple logistic regression to 0-100 points. The total score, which corresponds to the probability of a prediction, can be calculated by adding the scores of each variable. The prediction accuracy and consistency of the model are assessed using the calibration curve, receiver operating characteristic (ROC) curve, the area under the ROC curve (AUC), consistency index (C index), and ROC curve. The net benefits of the model to patients are reflected by decision curve analysis (DCA). By bootstrapping 1,000 resamples, identification and calibration are assessed.

### Web Application Development

To improve the practicability of this prediction, we have designed a network application for clinicians, that is, the predictor of IVH for ARDS newborns. This web tool is hosted on our server at http://43.143.217.126:8080/nice/.

### Statistical analysis

We use R software (version 4.1.2) 4.1.2 to process data and conduct statistical analysis. The Shapiro-Wilk test is used to determine whether data for continuous variables follow a normal distribution. Categorical variables are expressed as frequency and percentage, whereas continuous variables are expressed as mean ± standard deviation (SD). The measurement data of normal distribution or approximate normal distribution shall be subject to a t-test. Classified variables are adopted χ 2 test or Fisher exact test.

## Results

### Patients’ characteristics

**Fig. 1:**
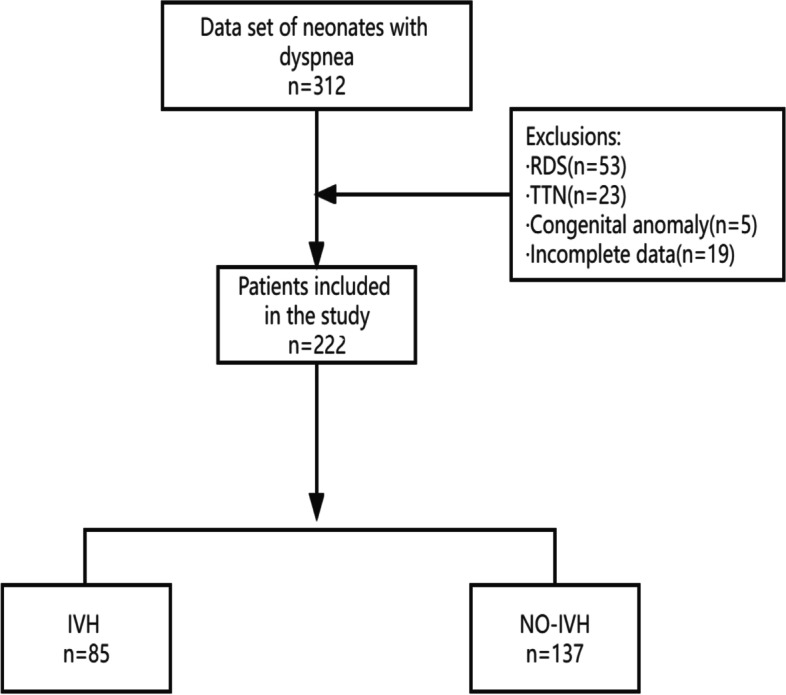
Flowchart of patient selection. ARDS = acute respiratory distress syndrome, TTN = transient tachypnoea of the neonate, IVH = intraventricular hemorrhage

Finally, 222 patients with ARDS were enrolled (Fig. 1), of which 70% were divided into training sets (*n* = 161) and the rest were verification sets (*n* = 61). The differences in all baseline data (neonatal characteristics, maternal characteristics, blood gas indicators, intervention) between the IVH group and NO-IVH group are shown in Table 1. IVH group had significant differences in gestational age, head circumference, length, premature rupture of membranes, multiple births, and PS dosage. In addition, the first blood gas analysis index data showed a significant difference between IVH and NO-IVH patients (*P* < 0.05).

**Table 1:** Baseline characteristics of patients

**Variables**	**Total** ***N*** **= 222**	**IVH (Grade I-II)** ***N* = 137**	**NO-IVH** ***N* = 85**	***p***
**Gender**				0.564
Male	116 (52.3%)	69 (50.4%)	47 (55.3%)	
Female	106 (47.7%)	68 (49.6%)	38 (44.7%)	
**GA(weeks)**				0.015
< 30	62 (27.9%)	48 (35.0%)	14 (16.5%)	
30–34 + 6	94 (42.3%)	55 (40.1%)	39 (45.9%)	
35–36 + 6	38 (17.1%)	18 (13.1%)	20 (23.5%)	
≥ 37	28 (12.6%)	16 (11.7%)	12 (14.1%)	
**BW (kilograms)**				0.258
< 1500	108 (48.6%)	72 (52.6%)	36 (42.4%)	
1500–2500	83 (37.4%)	49 (35.8%)	34 (40.0%)	
≥ 2500	31 (14.0%)	16 (11.7%)	15 (17.6%)	
**Length**	41.7 ± 5.31	41.0 ± 5.64	42.8 ± 4.52	0.009
**HC**	29.3 ± 2.69	28.9 ± 2.69	29.9 ± 2.59	0.010
**UC**				0.054
No	129 (58.1%)	87 (63.5%)	42 (49.4%)	
Yes	93 (41.9%)	50 (36.5%)	43 (50.6%)	
**PROM**				<0.001
No	144 (64.9%)	109 (79.6%)	35 (41.2%)	
Yes	78 (35.1%)	28 (20.4%)	50 (58.8%)	
**Multiple births**				0.005
No	156 (70.3%)	106 (77.4%)	50 (58.8%)	
Yes	66 (29.7%)	31 (22.6%)	35 (41.2%)	
**Apgar score**				0.525
＞7	135 (60.8%)	85 (62.0%)	50 (58.8%)	
4–7	76 (34.2%)	47 (34.3%)	29 (34.1%)	
≤ 3	11 (4.95%)	5 (3.65%)	6 (7.06%)	
**PS dosage**				0.002
＞100	189 (85.1%)	125 (91.2%)	64 (75.3%)	
≤ 100	33 (14.9%)	12 (8.76%)	21 (24.7%)	
**PS**				0.654
No	172 (77.5%)	108 (78.8%)	64 (75.3%)	
Yes	50 (22.5%)	29 (21.2%)	21 (24.7%)	
**PH**^**1**^	7.31 ± 0.08	7.34 ± 0.06	7.26 ± 0.07	<0.001
**PaO**_**2**_^**1**^	76.6 ± 23.9	82.8 ± 23.5	66.7 ± 21.1	<0.001
**PaCO**_**2**_^**1**^	38.6 ± 9.56	35.4 ± 8.14	43.7 ± 9.49	<0.001
**BE**^**1**^	-7.16 ± 2.96	-6.94 ± 2.98	-7.52 ± 2.91	0.157
**PH**^**2**^	7.35 ± 0.10	7.36 ± 0.10	7.35 ± 0.09	0.469
**PaO**_**2**_^**2**^	83.7 ± 21.6	85.0 ± 20.7	81.6 ± 23.0	0.268
**PaCO**_**2**_^**2**^	36.9 ± 37.8	33.5 ± 11.0	42.3 ± 59.3	0.181
**BE**^**2**^	-7.02 ± 3.30	-6.67 ± 3.21	-7.57 ± 3.37	0.050
**Invasive ventilation**				0.167
No	111 (50.0%)	63 (46.0%)	48 (56.5%)	
Yes	111 (50.0%)	74 (54.0%)	37 (43.5%)	

*GA* gestational age, *BW* Birth weight, *HC* Uterine contraction, *HC* Head circumference, *PROM*  premature rupture of membrane, *PS* pulmonary surfactant, PH^1^, PaO_2_^1^, PaCO_2_^1^ and BE^1^ represent the first blood gas analysis value, PH^2^, PaO_2_^2^, PaCO_2_^2^ and BE^2^ represent the second blood gas analysis value

### Variables selection

**Fig. 2:**
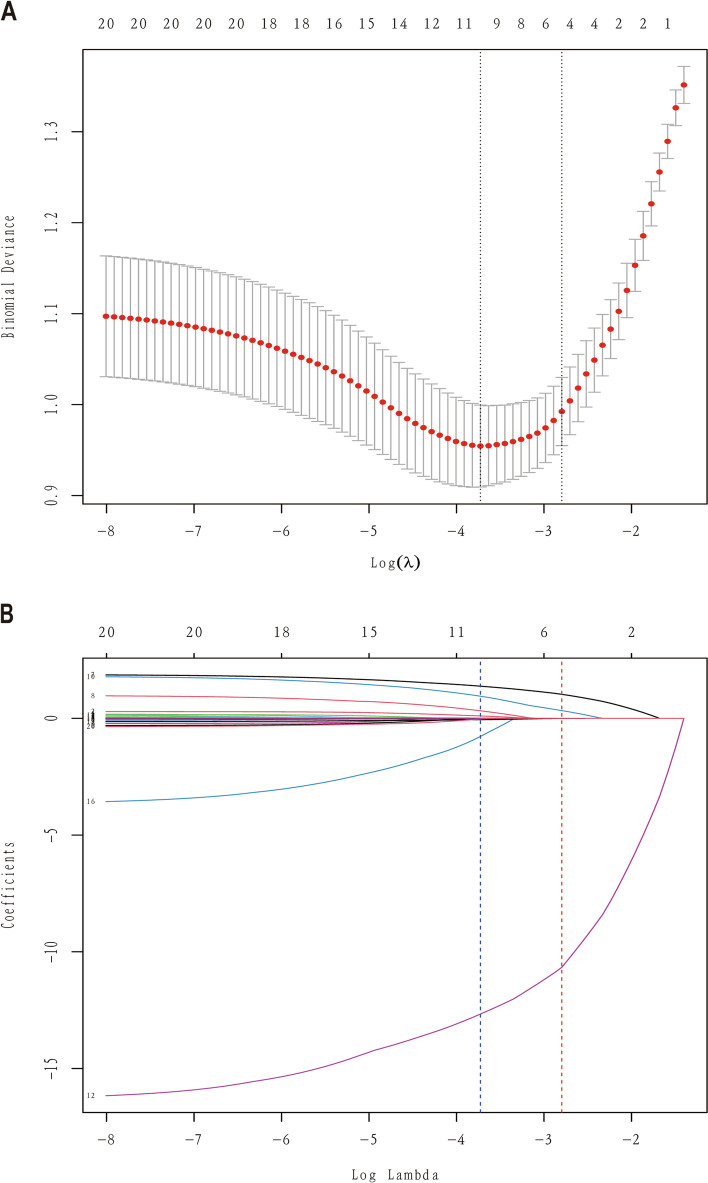
Feature selection based on LASSO binary logistic regression analysis. **A** The penalty coefficients in the LASSO model are adjusted using cross-validation and minimum criteria. **B** The prediction factors are determined by the cable regression method

In the LASSO regression model, the analysis results showed that 222 patients based on the non-zero coefficient cohort reduced 20 variables to 4 potential predictors through compression coefficient. (Fig. 2 A, B). These variables include PROM, PS dosage, PH^1^ and PaO_2_^1^ (Table 2).

**Table 2:** Predictors of IVH in ARDS neonates

**Characteristic**	**OR**	Predictors of IVH in ARDS neo	***p***
**PROM**			
No	—	—	
Yes	5.33	2.63, 11.2	< 0.001
**PS dosage**			
＞100	—	—	
≤ 100	2.21	0.82, 6.11	0.12
**PH**^**1**^	0.00	0.00, 0.00	< 0.001
**PaO**_**2**_^**1**^	0.97	0.96, 0.99	0.002
*OR* Odds Ratio, *CI* Confidence Interval, *PROM* premature rupture of membrane, *PS* pulmonary surfactant, PH^1^ and PaO_2_^1^ represent the first blood gas analysis value			

### Development of Nomogram

**Fig. 3:**
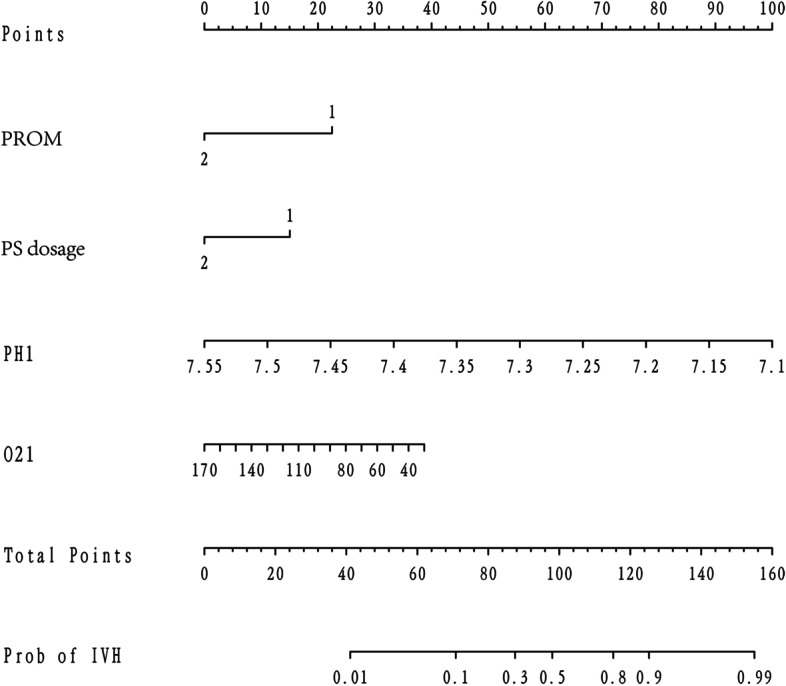
Nomogram of IVH prediction in newborns with ARDS. PROM = premature rupture of membrane, PS = pulmonary surfactant, PH^1^ and O_2_^1 ^respectively represent the PH value and oxygen partial pressure value of the first blood gas analysis

According to the results of multivariable logistic regression analysis, the following factors are related to IVH: PROM, PS dosage, PH^1^and PaO_2_^1^. These four factors were included in the prediction model, and a nomogram was formed to visualize the results of regression analysis **(**Fig. 3).

### Validation of nomogram

**Fig. 4:**
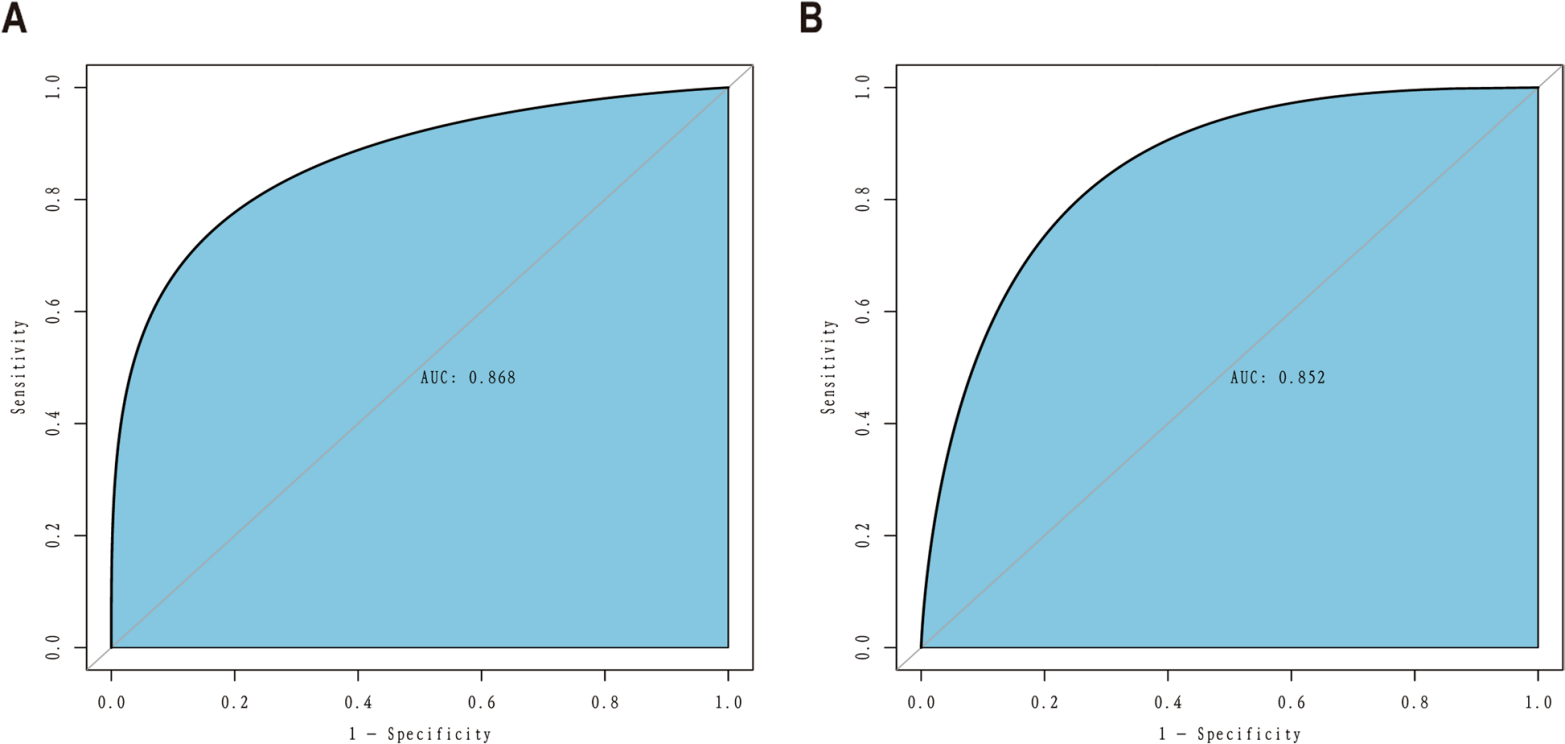
ROC curves. **A** Training cohort, **B** Validation cohort, ROC = receiver operating characteristic, AUC = area under the ROC curve

**Fig. 5:**
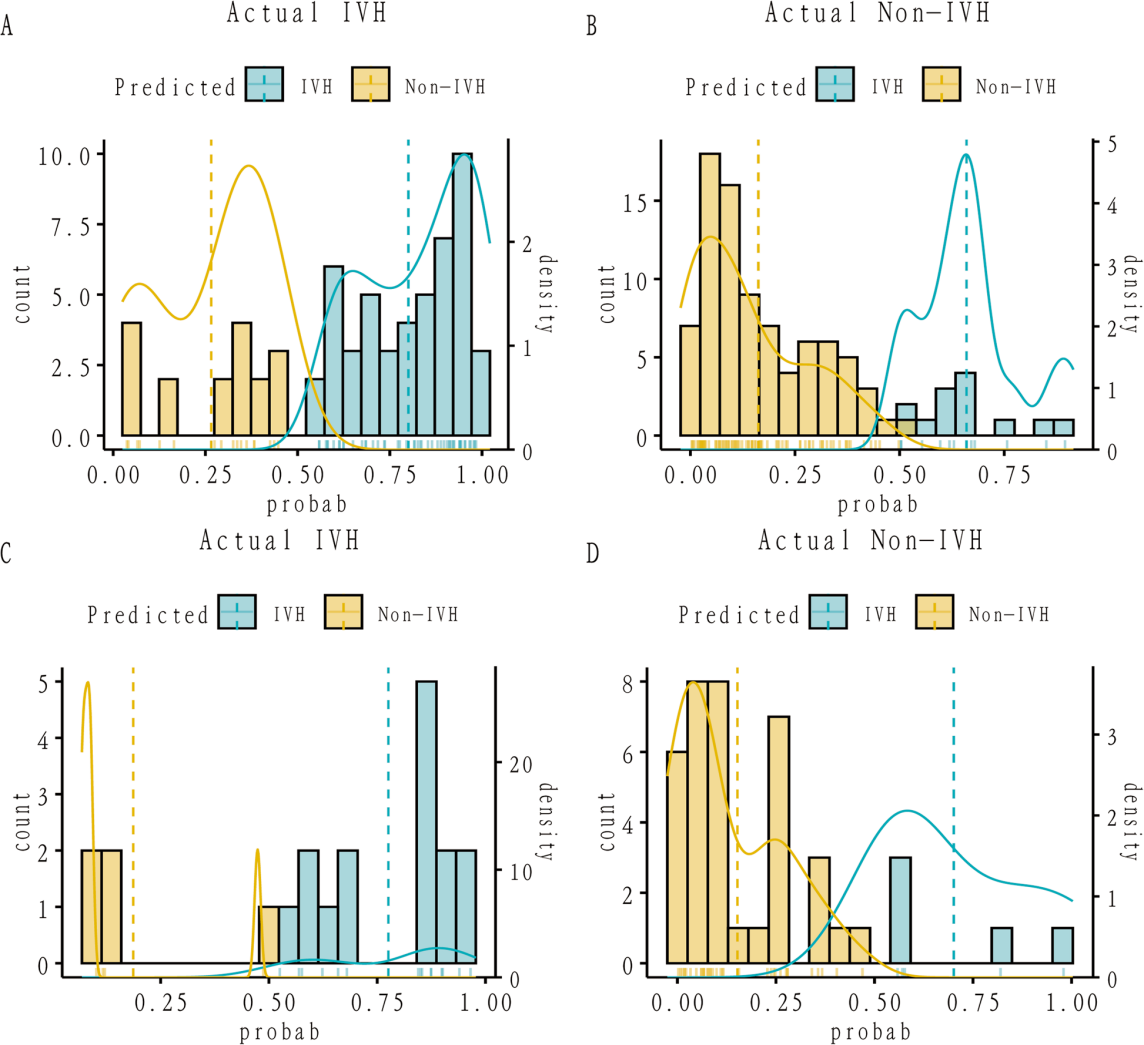
Case probability density plots (**A**) IVH prediction in training set, (**B**) NO-IVH prediction in training set, (**C**) IVH prediction in Validate set, (**D**) NO-IVH prediction in Validate set

**Fig. 6:**
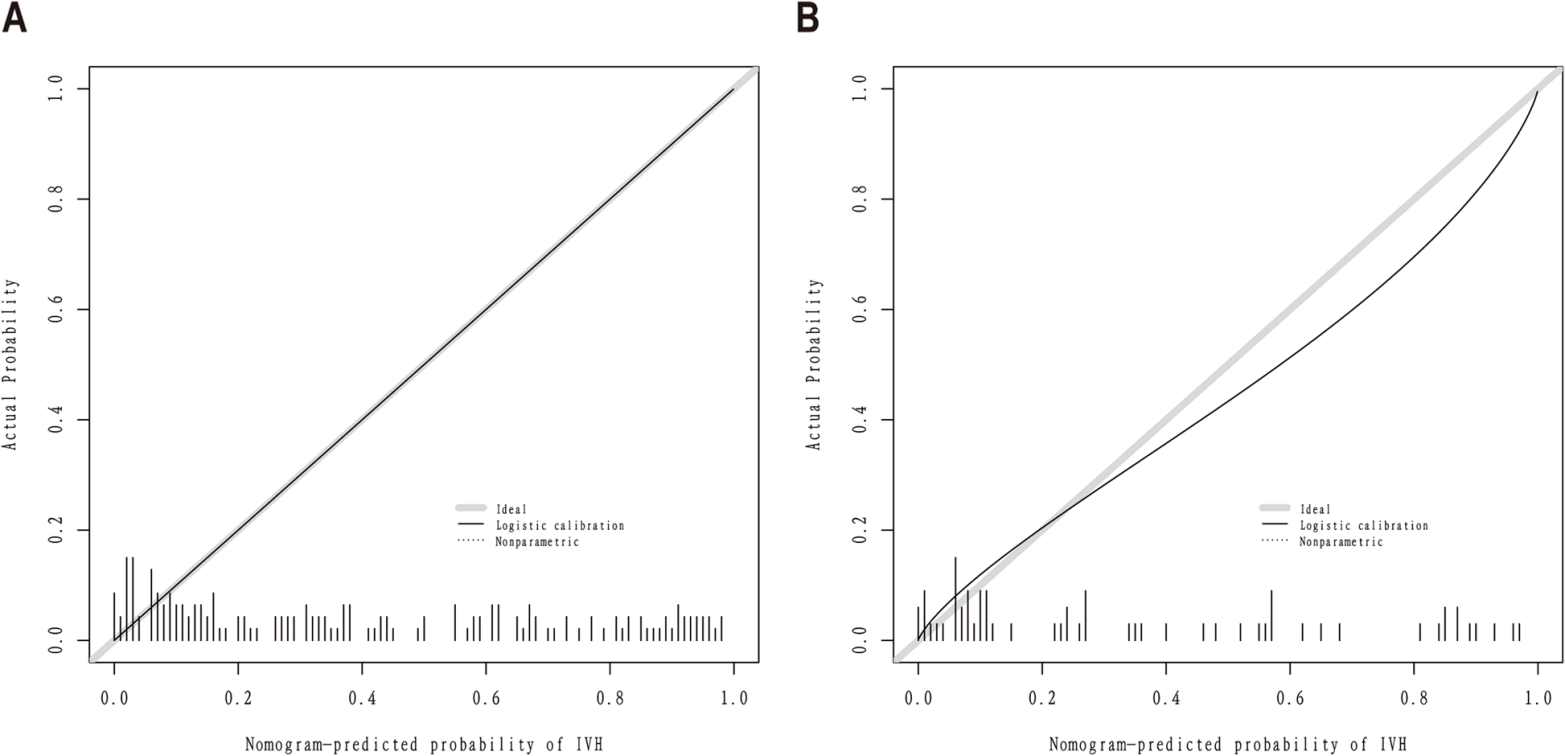
Calibration curve for predicting the probability of IVH in patients with ARDS. **A** Training cohort, **B** Validation cohort

The C-index for the prediction nomogram was 0.868 (95% CI: 0.837-0940) for the training cohort and 0.857 via bootstrapping validation **(**Fig. 4 A, B**)**, indicating the model’s good discrimination. And we used the Case probability density plots to more intuitively understand the distribution of decision probability (Fig. 5). It is helpful to compare the distribution of correct and wrong prediction probability of IVH and NO-IVH populations in the training set and validation set. Subsequently, the calibration curve of the predictive model used to assess the risk of IVH in patients with ARDS shows satisfactory consistency in this dataset (Fig. 6).

### Clinical application

**Fig. 7:**
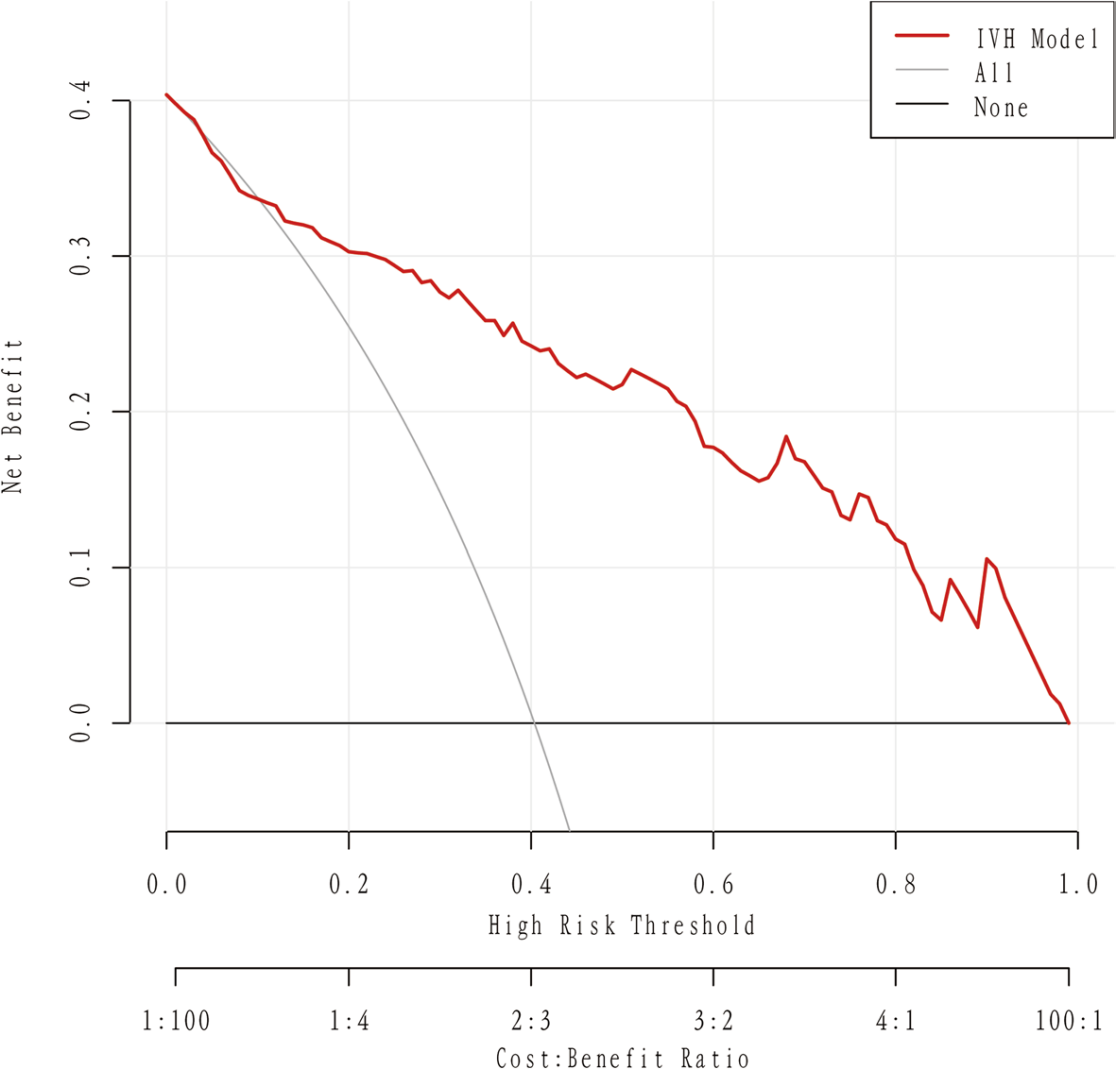
Nomogram decision curve for Intraventricular hemorrhage in the training cohort

Clinical application Decision analysis (DCA) was performed on the data to assess the clinical usefulness of the prediction model. This new method, called DCA, is used to evaluate the clinical net benefit of a nomogram. The analysis of the decision curve shows that the model can significantly improve clinical efficiency in predicting IVH, as shown in Fig. 7.

### Web Tool Development

**Fig. 8:**
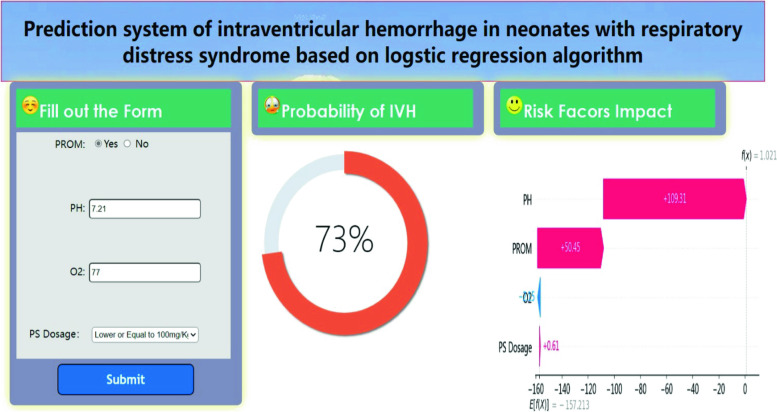
Web Application Screenshot

We have developed a free online network to use this web application in order to use this model for IVH prediction (Fig. 8). Just enter whether PROM occurs, PS usage dose, PH and PaO_2_^1^ values of the first blood gas analysis, and then click the “Submit” option. Then, the website will display the IVH risk score of the subject. This helps clinicians compare risks and develop individual treatment strategies.

## Discussion

IVH is the most common brain problem of premature infants, and its related mortality is still high [22], even in NICU operated by neonatal pediatricians. Previous studies have shown an increased risk of neurodevelopmental disorders in preterm infants with respiratory distress syndrome [23]. Several other studies also support that ARDS is a risk factor for severe IVH [24, 25]. The purpose of this study was to identify variables associated with IVH in a newborn sample of ARDS. For children with ARDS, early prevention of IVH is very important because IVH not only reduces the survival rate of newborns, but also increases the risk of many neurological sequelae. Premature infants with moderate and severe IVH are at high risk of cerebral palsy and mental retardation, while newborns with mild IVH are at risk of developmental disorders [26]. Other studies have shown that premature infants with grade I and II low-degree intraventricular hemorrhage may have abnormal neurodevelopmental results [27, 28]. It shows that even different levels of IVH may cause serious neurological complications. With the development of neonatal intensive care unit, the neonatal incidence rate of IVH survivors has changed from severe cerebral palsy to mild neurocognitive impairment [29, 30]: the incidence of mild cerebral palsy has increased while the incidence of severe cerebral palsy has decreased [31]. This also shows that the focus of people’s attention has slowly shifted to the possible mild nervous system development disorder. For children with cerebral hemorrhage, a few of these infants may exhibit subtle abnormalities in consciousness, movement, respiration, or eye movements, but the majority of them do not exhibit any symptoms. This also provides difficulties for early identification. Moreover, bleeding is likely to progress. Wu et al. [32] showed that 8.2% of Class II/III GM-IVH premature infants (< 32 weeks) deteriorated to Class II/IV GM-IVL within 7 days. Therefore, early screening of high-risk neonates is of great significance to guide clinicians in treatment and prevention.

IVH usually starts in the germinal matrix, which is a collection of blood vessels rich in glial precursor cells in the developing brain. Although the cause of neonatal brain injury is multifactorial, cerebral blood flow (CBF) [33] and cerebral autoregulation disorder [34] play an important role. Numerous prenatal, perinatal, and postpartum factors have been confirmed as separate risk factors for neonatal IVH. These include inflammation, acidosis, hypoxemia, lack of maternal prenatal steroid management, chorioamnionitis, multiple pregnancies, etc. Additional risk factors include pulmonary surfactant use and clinically significant mechanical ventilation [35].

Our study is the first to use a nomogram to predict the risk of IVH in newborns with ARDS based on four clinically relevant variables. Good identification and calibration capabilities are demonstrated by the internal validation analysis. PROM, PH^1^, PaO_2_^1^ and PS < 100 mg/kg were found to be independent risk factors for IVH in ARDS in our study. PROM, low dose PS, low PH and hypoxia can increase the risk of IVH. PROM, especially during delivery within 24 h, is often associated with intrauterine infection [36]. According to research, babies born to mothers with premature rupture of membranes are more likely to have severe IVH than those born to mothers with hypertension [37]. The intrauterine infection leads to the production of various inflammatory factors, which can damage astrocytes, oligodendrocytes and white matter of the brain and cause brain damage [38]. Inflammatory factors will be released when inflammation occurs in the pregnant mother or intrauterine infection occurs in the fetus, which will activate microglia in the central nervous system, mediate the damage and even kill surrounding cells such as neurons or glial cells.

Pulmonary surfactant has been proven to be an indication for patients with ARDS. When neonates have dyspnea or respiratory failure, early use of pulmonary surfactant can alleviate the symptoms of dyspnea, reduce the degree of hypoxic-ischemic injury, maintain the stability of cerebral hemodynamics, and reduce the occurrence of IVH. The clinical multicenter study on preterm infants less than 27 weeks gestation confirmed that the use of a non-invasive surfactant application (LISA) strategy in the early postpartum period without invasive ventilation can significantly reduce the incidence of severe IVH [39]. Mild respiratory distress syndrome associated with postpartum surfactant therapy is associated with decreased IVH [40, 41].

Brain injury and brain dysfunction in premature infants are related to hypoxia, hypercapnia and acidemia [42]. Blood gas disorder is more likely to cause brain damage than other factors [43]. Hyperserotonemia may affect the development of inferior vena cava blood flow by causing vasodilatation of cerebral resistant arterioles, increasing CBF and impairing complete cerebral autoregulation [44, 45]. In addition, hypercapnia may cancel or damage the self-regulation of the brain, leading to the risk of ischemia or over-perfusion of the cerebrovascular system during blood pressure fluctuations. Ipsita R. Goswami et al. showed that metabolic acidosis in preterm infants within 72 h after birth is related to IVH, rather than low/high carbonation [46]. Higher acidity levels within 48 h after birth are associated with an increased incidence of IVH [13]. We consider that acidosis may be closely related to brain autoregulation disorder, which may cause early brain damage.

In this study, the incidence of IVH in children with ARDS is 38.2%, which is higher than the incidence of premature infants reported in pertinent literature [11, 47]. It is considered to be related to this study object. Moreover, in our study, the incidence rate of moderate to severe (3–4 grades) IVH is extremely low. We consider that this may be related not only to the time limit of our ultrasound inclusion, but also to the low incidence rate of severe IVH [48]. In order to facilitate the universality of the model, we did not include children with moderate to severe IVH. Recent evidence suggests that any degree of bleeding may be associated with abnormal neurodevelopmental results, although adverse results are usually associated with severe GM-IVH [7, 9, 10]. However, premature infants previously diagnosed as mild IVH may deteriorate to severe GM-IVH. Therefore, this study focuses on early identification of mild IVH children and providing positive preventive measures for later progress. In the future, it may be necessary to carry out a multi-center large sample size study to further include children with IVH at different levels.

We screened out risk factors according to the prenatal and perinatal conditions of previous patients. We developed a perinatal prediction nomogram for the early prediction of IVH in neonatal ARDS based on these perinatal predictors. Our internal validation confirmed that the model was reliable. The higher overall score for each patient reflects the increased risk of IVH in those with ARDS. This visual prediction model provides clinicians with an easy-to-use tool for early identification of IVH in newborns with ARDS, which may be of great significance in reducing the corresponding complications of IVH. We found that the IVH nomograph decision curve in the training cohort suggested the feasibility of clinical use. By paying attention to PROM patients and conducting blood gas testing at an early stage, and then immediately assessing the IVH risk of children with ARDS according to our model, we can distinguish between high-risk groups and low-risk groups. According to our model, the risk of IVH in children with ARDS was assessed immediately after birth to distinguish high-risk and low-risk populations. After birth, high-risk newborns should fully inhale oxygen, especially avoid hypoxia and acidosis, maintain cerebral blood flow without obvious interference, and reduce the occurrence of IVH. If it is necessary to inject pulmonary surfactant, pay attention to adequate use, and conduct regular blood gas analysis. However, for children with low risk, follow-up observation is also required. This model can be used to determine which type of infant is recommended to improve ultrasound for clear diagnosis. In order to reduce the severity of bleeding through timely and early intervention. It will become a convenient tool for primary hospitals to identify high risks as early as possible and implement appropriate intervention measures, while also avoiding over-treatment of low-risk patients.

This study has some limitations. The model is validated internally, but external validation using data from other regions or hospitals will be more rigorous. We collected and analyzed the data retrospectively, which may lead to deviation. In addition, we only considered children who had undergone blood gas testing and ultrasound testing and excluded a few newborns with relatively benign clinical courses. In addition, pregnancy age is an important factor affecting ARDS. However, due to the strict inclusion and exclusion criteria of this study and the fact that our ward is a provincial medical center, there is a selection bias in the patient registration in our cohort, so the statistical results of gestational age are not significant, so they are not included.

## Conclusion

In conclusion, we investigated the relationship between IVH and predictors: PROM, PS dosage, PH^1^ and PaO_2_^1^. Our nomographs performed well in the assessment of IVH, which can help clinicians make clinical decisions and determine whether patients with ARDS are at risk of developing IVH according to the nomographs. However, external verification is still required in the future.

## Data Availability

The data set generated or analyzed in this study can be obtained from the first author and corresponding author according to reasonable requirements.
